# Small Centers with Big Ventures: Autologous Stem Cell Transplantation Survival Data

**DOI:** 10.31557/APJCP.2019.20.4.987

**Published:** 2019

**Authors:** Muhammad Bilal Abid, Mustafa Mughal, Ranjeet Babbra, Muhammad Abbas Abid, Norbert Blesing, Saiyed Anwer

**Affiliations:** 1 *Department of Haematology, Great Western Hospitals NHS Trust, Swindon, *; 3 *Department of Haematology, University Hospitals of Bristol NHS Trust, UK,*; 2 *Department of Medicine, Medical College of Wisconsin, Milwaukee, WI,*; 5 *Department of Otolaryngology-Head and Neck Surgery, Johns Hopkins University School of Medicine, Baltimore, MD, USA,*; 4 *Department of Internal Medicine, Liaquat College of Medicine and Dentistry, Karachi, Pakistan. *

**Keywords:** Outcome analysis, autologous stem cell transplantation, district general hospital, secondary care center

## Abstract

We present the first-ever autologous stem cell transplantation (ASCT) outcome data from a secondary-care healthcare facility. Albeit exact details of patient and disease characteristics and co-morbidity scores for all patients are not available, the engraftment and survival data is very similar to those published from large tertiary-care cancer centres, both regionally and internationally. Transplant Related Mortality (TRM) of 3.1% is within the expected range and includes a patient who died of acute drug reaction (ADR) during conditioning chemotherapy, prior to the ASCT. Furthermore, cyclophosphamide mobilization chemotherapy is given in the outpatient setting. This study is important in terms of healthcare resource optimization as well as patients’ convenience and highlights that ASCT can be performed in a safe and effective manner with comparable survival rates even at a DGH, provided the centre stays abreast with the recent developments and can offer its patients with standard of care treatment of the era.

## Introduction

Autologous stem cell transplantation (ASCT) remains the leading method of bone marrow transplantation (BMT), both in the upfront and in the salvage settings, either with a curative-intent or as a survival-prolonging strategy for multiple myeloma (MM) and lymphoma. Child et al., (2003) demonstrated that high-dose therapy (HDT) followed by ASCT prolongs survival in transplant-eligible MM patients, whereas this strategy holds potential for cure for a fraction of patients with relapsed/refractory Hodgkin’s (HL) and Non-Hodgkin’s Lymphoma (NHL) (Gerrie et al., 2014; Philip et al., 1995). 

Since the stem cells are derived from the same individual, ASCT carries a superior morbidity and mortality profile in comparison with allogeneic transplant. There are fewer acute and chronic transplant-related complications and no graft-vs-host disease (GVHD), albeit at the expense of higher relapse rate (Pasquini and Wang, 2013). There are also less healthcare resources consumed and, hence, it is being performed both in the inpatient as well as outpatient settings globally (Martino et al., 2016; Abid et al., 2017). In addition, ASCT may yet rarely be performed at the secondary-level healthcare centres which may house facilities and manpower to perform ASCT and adhere to international standards [The Foundation for the Accreditation of Cellular Therapy (FACT) and the Joint Accreditation Committee – ISCT and EBMT (JACIE); (FACT-JACIE Standards, 6th Edition)]. Great Western Hospital (GWH) in the southwest city of Swindon, England is one such secondary-level centre which is JACIE-accredited and perform ASCT regularly. 

It would be enormously convenient for patients to receive this potentially curative, state-of-the-art treatment within their cities/towns or in the vicinity and not have to travel several hundred miles. Though ASCT is being performed in secondary-care centres in Western countries, no long-term ASCT outcomes data is available from them. The purpose of this study was to learn if ASCT outcomes performed at a district general hospital (DGH) in the United Kingdom, equivalent to a secondary-care centre, are any different from those performed at large, tertiary care cancer centres. The outcome variables presented are engraftment, transplant-related mortality (TRM), overall survival (OS) and progression-free survival (PFS). We further present general trends observed during the 18-year study period. 

## Materials and Methods

This was a retrospective review of 129 consecutive patients at GWH, between January 1998 and December 2015. All patients underwent ASCT as per the EBMT (European Society for Blood and Marrow Transplantation) criteria. For PBSC mobilisation, MM patients received cyclophosphamide 3,000mg/m^2^ whereas those with lymphoma received either ESHAP (Etoposide 40mg/m^2^, Methylprednisolone 500mg daily from day 1 through day 5, Cytarabine 2,000mg/m^2^ and Cisplatin 25mg/m^2^) or ICE (Ifosfamide 5,000mg/m^2^, Carboplatin AUC of 5 [maximum dose 800mg/m^2^] and Etoposide 100mg/m^2^ on days 1, 2 and 3), both with or without Rituximab 375 mg/m^2^. After the peripheral blood stem cell (PBSC) count of >/= 10 x 10^6^/L was achieved, patients were sent to National Health Services Blood and Transplant (NHSBT) centre at the Oxford University Hospital (OUH), located 30 miles northeast of Swindon, for stem cell apheresis. The harvested cells were then processed and stored at OUH and were requested a week prior to the infusion day. PBSCs were transported to the patient’s room in a dry shipper, in the vapour phase of liquid nitrogen, and the temperature of the water bath was consistently maintained below 40°C. DMSO toxicity could be reduced by limiting infusion to 1ml DMSO/kg of recipient weight/day; the equivalent of no more than 10mls of thawed cells/kg/day (using 10% DMSO). PBSC infusion was then performed after 24 hours of myeloablative chemotherapy.

Apheresis was continued until at least 6 × 10^6 ^CD34+ PBSC/kg of body weight (BW) were collected. All patients achieved target stem cell dose. Patients who proceeded to HDT after successful PBSC collection received conditioning with BEAM (BCNU/Carmustine, Etoposide, Cytarabine, Melphalan) or melphalan, either full dose of 200mg/m^2^ or intermediate dose of < 200mg/m^2^. PBSC infusion was performed on D0 with a minimum CD34 + PBSC dose of 2 x 10^6^/kg BW. Standard anti-infective prophylaxis and supportive care were rendered in the peri- and post-ASCT period. Anti-infective prophylaxis in the peri-ASCT setting included acyclovir and fluconazole whereby acyclovir was continued until 90 days, post-transplant. In the post-ASCT period, trimethoprim/sulfamethoxazole was continued till 120 days post-transplant and ciprofloxacin until the first 14 days, at a minimum. We retrieved data on patient demographics (age and gender), ASCT trends across 2 decades, haematological disorders during the study period, engraftment: stratified according to conditioning regimen used, TRM: defined by death within 100 days of ASCT, and OS and PFS: stratified according to transplanted disorders. Data was collected via homogenized EBMT registry via Minimal Essential Data-A (MED-A) forms onto spreadsheets. Data was analyzed and survival curves were generated using the Winstat software.

## Results

Over the last decade, the advent of newer chemo/immunotherapeutic agents has changed the entire outlook of haematological malignancies. Novel agents have substantially prolonged survival of MM whereas Rituximab-based combination chemotherapy offered better cure rates for B-cell NHL (Brenner et al., 2008). GWH has also kept abreast with developments and has rendered its patients with the standard-of-care treatment, either through National Health Service (NHS), industry or via compassionate drug fund (CDF). Since the first ASCT in 1998, GWH has performed more ASCT in males (74/129; 57.4%) than in females (55/129; 42.5%). The median age was slightly younger in males (53.7 vs 55.3) and the median age at ASCT, regardless of the disorder, was 57.3 (19-71), which increased over the study period. The median number of ASCT performed was 6.5, meeting the JACIE accreditation standard of a minimum of 5 ASCT procedures per annum (FACT-JACIE, 2015). This progressively increased over the 18-year period with the least number of transplants performed in 1998 (2) and the most in 2015 (16). MM, Diffuse Large B-Cell Lymphoma (DLBCL) and Hodgkin’s Lymphoma (HL) accounted for the majority of ASCT with other NHL subtypes accounting for the rest. The median ages in different disease subgroups are depicted in Table 1. 

**Figure 1:**
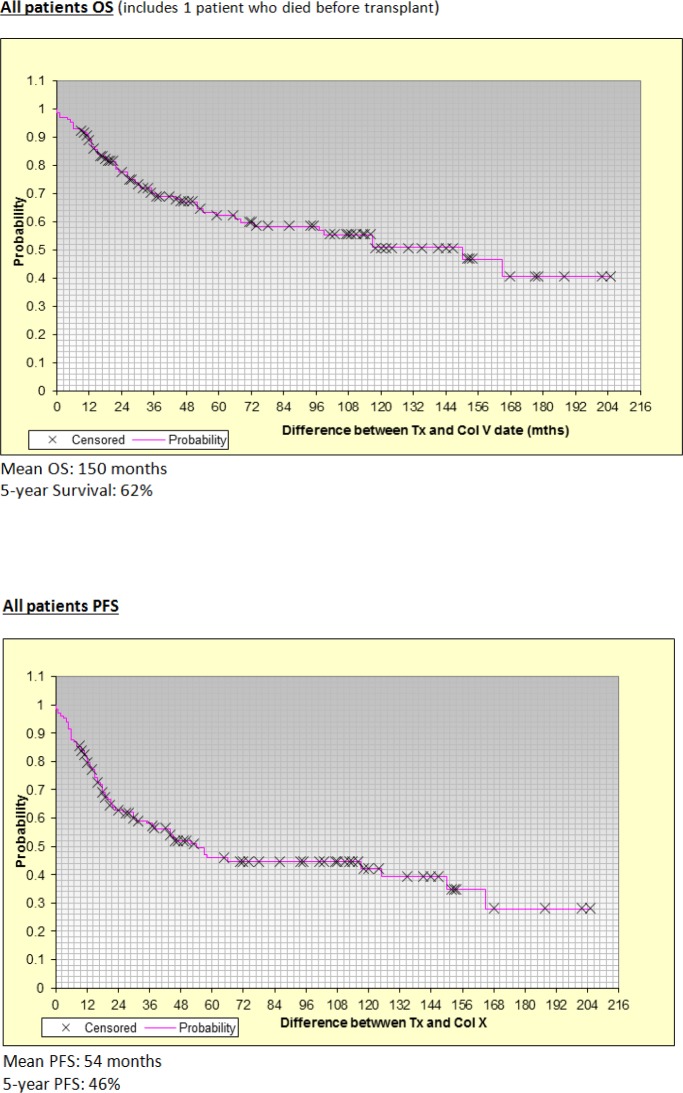
Overall Survival (OS) and Progression Free Survival (PFS) Data

**Table 1:** Median Age of Patients and Proportion of Haematological Disorders That Underwent ASCT

Disease	Median Age	# of patients	percentage
MM	59.7	55	43
DLBCL	56.8	42	33
HL	36.2	16	12
T-Cell NHL	41.5	6	5
FL	51.9	5	4
MCL	55.6	4	3
WM	66.5	1	1

MM, Multiple Myeloma; DLBCL, Diffuse Large B Cell Lymphoma; HL, Hodgkin’s Lymphoma; NHL, Non-Hodgkin’s Lymphoma; FL, Follicular Lymphoma; MCL, Mantle Cell Lymphoma; WM, Waldenstrom Macroglobulinaemia.

**Table 2:** Dose of CD34+ PBSC Infused and Count Recovery, for BEAM and the 2 Cohorts of Melphalan

	BEAM (n = 74)	MEL 200mg/m2 (n =43)	MEL <200mg/m^2^ (n =12)
CD34+ PBSC dose infused	5.9 x 106/kg	4.5 x 106/kg	
Median ANC Recovery (Range)	13 (8-28) *	13 (10-25)	12 (10-25)
Median Platelet Recovery (Range)	27 (10-425) **	25 (11-70)	24 (12-54) ***

* 2 patients, No ANC engraftment; ** 4 patients, No platelet engraftment; *** 1 patient, No platelet engraftment.

BEAM was used in 57.4% of patients conditioning regimen whereas melphalan was used in the remaining 42.5%, either in full or reduced dose. All patients but 2 did not achieve neutrophil engraftment and both had received BEAM conditioning. 5 patients did not accomplish platelet engraftment, 4 from the BEAM arm and 1 from the intermediate-dose melphalan cohort. The engraftment data is shown in Table 2. Local institutional guidelines were followed in cases where patients did not engraft. There were 4 deaths within the first 100 days of ASCT, resulting in a TRM of 3.1%. Of those 4 patients, 2 died of sepsis with infection (one due to pneumonia with parainfluenza virus and other with bacteraemia), 1 died of lymphoma relapse and another due to acute drug reaction leading to respiratory failure. Mean OS was 150 months whereas 5-year OS was 62%. Mean PFS was 54 months whereas 5-year PFS was 46%. Mean OS and PFS for all patients are shown in Figure 1. 

## Discussion

Although retrospective, we herein present the first-ever ASCT outcome data from a secondary-level healthcare facility. Albeit exact details of patient, disease characteristics and co-morbidity scores for all patients are not available, the engraftment and survival data are very similar to those published from large tertiary-care cancer centres, both regionally and internationally (D’ Souza et al., 2017; Ali et al., 2015; Auner et al., 2015). TRM of 3.1% is within the expected range and includes a patient who died of acute drug reaction during conditioning chemotherapy, prior to the actual ASCT. Furthermore, cyclophosphamide mobilisation chemotherapy is given in the outpatient setting. 

In conclusion, this study is important in terms of healthcare resource optimization as well as patients’ convenience and highlights that ASCT can be performed in a safe and effective manner with comparable survival rates even at a DGH, provided the centre stays abreast with the recent developments and can offer its patients with standard of care treatment of the contemporary era.

## Conflict of interest

The authors declare no competing financial interests to disclose for this study.
